# Red Cell Microparticles Suppress Hematoma Growth Following Intracerebral Hemorrhage in Chronic Nicotine-Exposed Rats

**DOI:** 10.3390/ijms232315167

**Published:** 2022-12-02

**Authors:** Ashish K. Rehni, Sunjoo Cho, Zhexuan Zhang, Priyanka Khushal, Ami P. Raval, Sebastian Koch, Miguel A. Perez-Pinzon, Weizhao Zhao, Wenche Jy, Kunjan R. Dave

**Affiliations:** 1 Peritz Scheinberg Cerebral Vascular Disease Research Laboratories, Miller School of Medicine, University of Miami, Miami, FL 33136, USA; 2 Department of Neurology (D4-5), Miller School of Medicine, University of Miami, Miami, FL 33136, USA; 3 Department of Biomedical Engineering, University of Miami, Coral Gables, FL 33146, USA; 4 Neuroscience Program, Miller School of Medicine, University of Miami, Miami, FL 33136, USA; 5 The Wallace H Coulter Platelet Laboratory, Division of Hematology/Oncology, Department of Medicine, Miller School of Medicine, University of Miami, Miami, FL 33136, USA

**Keywords:** cigarette, e-cigarette, tobacco, smoking, risk factor, stroke

## Abstract

Spontaneous intracerebral hemorrhage (sICH) is a disabling stroke sub-type, and tobacco use is a prominent risk factor for sICH. We showed that chronic nicotine exposure enhances bleeding post-sICH. Reduction of hematoma growth is a promising effective therapy for sICH in smoking subjects. Red-blood-cell-derived microparticles (RMPs) are hemostatic agents that limit hematoma expansion following sICH in naïve rats. Considering the importance of testing the efficacy of experimental drugs in animal models with a risk factor for a disease, we tested RMP efficacy and the therapeutic time window in limiting hematoma growth post-sICH in rats exposed to nicotine. Young rats were chronically treated with nicotine using osmotic pumps. sICH was induced in rats using an injection of collagenase in the right striatum. Vehicle/RMPs were administered intravenously. Hematoma volume and neurological impairment were quantified ≈24 h after sICH. Hematoma volumes in male and female nicotine-exposed rats that were treated with RMPs at 2 h post-sICH were significantly lower by 26 and 31% when compared to their respective control groups. RMP therapy was able to limit hematoma volume when administered up to 4.5 h post-sICH in animals of both sexes. Therefore, RMPs may limit hematoma growth in sICH patients exposed to tobacco use.

## 1. Introduction

Spontaneous intracerebral hemorrhage (sICH) refers to a condition characterized by bleeding in the brain parenchyma or ventricular system occurring due to factors other than trauma [1]. sICH is a deadly form of hemorrhagic stroke, which is responsible for as much as 28% of stroke cases worldwide [2]. One-month mortality associated with sICH is approximately 40% [3,4]. Tobacco use is a prominent risk factor for sICH [5]. Smoking is prevalent in young sICH patients [6]. Moreover, cigarette smoking causes increased hematoma volume, worse outcomes, and increased mortality following sICH [7]. Our group has recently shown that chronic exposure to nicotine increases hematoma expansion after sICH [8].

Hematoma size increases over several hours after the onset of sICH [9], and hematoma size correlates with mortality [3]. Limiting hematoma growth appears to be a logical therapeutic strategy for sICH [10]. Some hemostatic agents have been tested for their potential efficacy in sICH, but they were either not found to be effective or associated with adverse events [11,12,13]. Thus, there is a need to identify safer hemostatic agents that can more effectively limit bleeding in the brain of patients suffering from sICH [14].

Previously, our group has shown that red-blood-cell-derived microparticles (RMPs) enhance both primary and secondary hemostasis and thus act as a universal hemostatic agent [15]. Microparticles are membrane fragments that expose the pro-coagulant phospholipid phosphatidylserine [16,17] that serves as a catalytic surface for the complex formation of clotting factors [17]. Our previously published data show that RMP treatment lowers the rate of bleeding in experimental models, and the presence of RMPs accelerates blood coagulation in vitro in blood from normal human subjects as well as subjects suffering from coagulation disorders [15]. Moreover, we have previously optimized the dose and treatment regimen of RMPs that effectively limits hematoma growth and functional deficits in naïve rats post-sICH [18]. We also observed that RMP treatment effectively limits hematoma size when initiated as late as 4.5 h post-sICH. Further, we noted that RMP treatment exerts long-lasting protective effects in terms of decreasing sICH-related behavioral impairment as well as lesion size in the brain of naïve rats [18].

However, the efficacy of RMPs in limiting hematoma expansion following sICH has not been tested in animals with risk factors for sICH. Since tobacco use is a significant risk factor [5,19,20], we tested the potential efficacy of RMPs in limiting hematoma expansion and neurological impairment following sICH in a rat model of tobacco exposure. Chronic nicotine infusion using osmotic pumps in rats was used as a surrogate for tobacco use. The collagenase-induced model of sICH was employed in the present study as the hematoma in this model grows over time, as seen in sICH patients [21].

## 2. Results

### 2.1. Physiological Parameters

Physiological parameter data monitoring was carried out during the surgery, and levels were maintained in a normal range (Table 1, Table 2, Table 3 and Table 4). For most of the data, we did not notice substantial differences among the treatment groups (Table 1, Table 2, Table 3 and Table 4). We observed statistically significant differences in some of the comparisons. However, the extent of these differences is not expected to make a physiological impact on the sICH outcomes.

### 2.2. Stages of the Estrous Cycle

Nine animals were in estrus/proestrus stages and one animal was in the diestrus/metestrus stage in the vehicle-treated group of the RMP efficacy experiment. Similarly, in the RMP-treated group of the RMP efficacy experiment, nine animals were in the estrus/proestrus stages and one animal was in the diestrus/metestrus stage. Fourteen animals were in the estrus/proestrus stages and one animal was in the diestrus/metestrus stage in the vehicle-treated group of the therapeutic window experiment. For both 4.5 and 6 h post-sICH RMP treatment groups, nine animals were in the estrus/proestrus stages and one animal was in the diestrus/metestrus stage. The distribution of rats at different stages of estrous cycle between different groups in the efficacy and therapeutic window experiment were not statistically different, and the groups were balanced in terms of the proportion of animals at different stages of the estrous cycle.

### 2.3. RMP Treatment Limited Hematoma Growth Following sICH in Nicotine-Exposed Rats of Both Sexes

**Figure 1:**
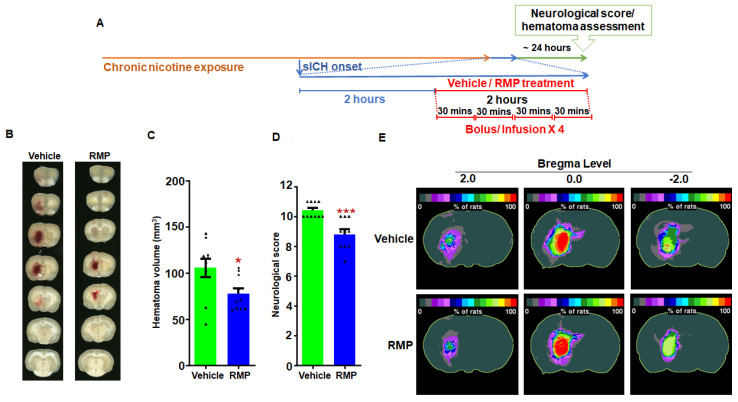
The effect of RMP treatment on hematoma expansion post-sICH in nicotine-exposed male rats: (**A**) representation of experimental design; (**B**) example images of brain scans showing mean hematoma volume; (**C**) mean hematoma volume data; (**D**) mean neurological score data; and (**E**) hematoma frequency maps at 3 coronal levels (bregma +2.0 mm, 0.0, and −2.0 mm). The top row depicts the hematoma frequency maps of vehicle-treated rats, and the bottom row shows the hematoma frequency maps of RMP-treated rats. *N* = 10 for vehicle- and RMP-treated groups, respectively; * *p* < 0.05 and *** *p* < 0.001 vs. vehicle group.

Smoking increases the risk of and worsens outcomes of sICH in human subjects [5,7]. We have previously shown that RMPs limit hematoma volume in naïve rats [18]. Therefore, we tested the hypothesis that RMP treatment limits chronic nicotine exposure-induced increase in bleeding following sICH in male and female rats. The total hematoma volumes (mm^3^) for the vehicle- and RMP-treated young male groups were 106 ± 10 (*n* = 10) and 78 ± 6 (*n* = 10), respectively (Figure 1B,C). The hematoma volume was 26% (*p* < 0.01) lower in the RMP-treated male rats when compared to the vehicle-treated rats. The hematoma frequency maps also show that RMP-treated nicotine-exposed male rats had smaller hematomas at multiple coronal levels in comparison to their vehicle-treated counterparts (Figure 1E). The neurological score in the RMP-treated male rats (7.4 ± 0.6) was significantly (*p* < 0.05) lower compared to the vehicle-treated control rats (9.3 ± 0.6) (Figure 1D). 

**Figure 2:**
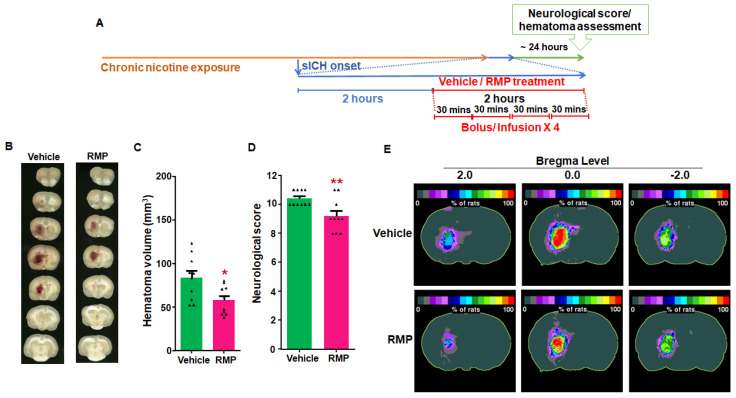
The effect of RMP treatment on hematoma expansion post-sICH in nicotine-exposed female rats: (**A**) representation of experimental design; (**B**) example images of brain scans showing mean hematoma volume; (**C**) mean hematoma volume data; (**D**) mean neurological score data; and (**E**) hematoma frequency maps at 3 coronal levels (bregma +2.0 mm, 0.0, and −2.0 mm). The top row depicts the hematoma frequency maps of vehicle-treated rats, and the bottom row shows the hematoma frequency maps of RMP-treated rats. *N* = 10 for vehicle and RMP-treated groups, respectively; * *p* < 0.05 and ** *p* < 0.01 vs. vehicle group.

The hematoma volume (mm^3^) observed in the vehicle- and RMP-treated young female groups were 84 ± 8 (*n* = 10) and 58 ± 5 (*n* = 10), respectively (Figure 2B,C). The hematoma volume in RMP-treated female rats was 31% (*p* < 0.05) lower than in vehicle-treated control rats. RMP-treated rats demonstrated a smaller hematoma volume than the vehicle-treated control rats when compared in terms of the hematoma frequency maps (Figure 2E). Further, the neurological score observed in RMP-treated female rats (7.4 ± 0.6) was significantly (*p* < 0.01) lower compared to the control rats (9.3 ± 0.6) (Figure 2D). These results show that RMP treatment limits hematoma expansion as well as improving neurological deficits post-sICH in the nicotine-exposed rats of both sexes. This signifies that, in line with our previous findings showing efficacy in naïve rats, RMPs also exert their hemostatic effect on sICH-induced bleeding in an animal model of tobacco use.

### 2.4. RMP Therapy Limited Hematoma Expansion When Administered as Late as 4.5 h Post-sICH in Nicotine-Exposed Rats of Both Sexes

**Figure 3:**
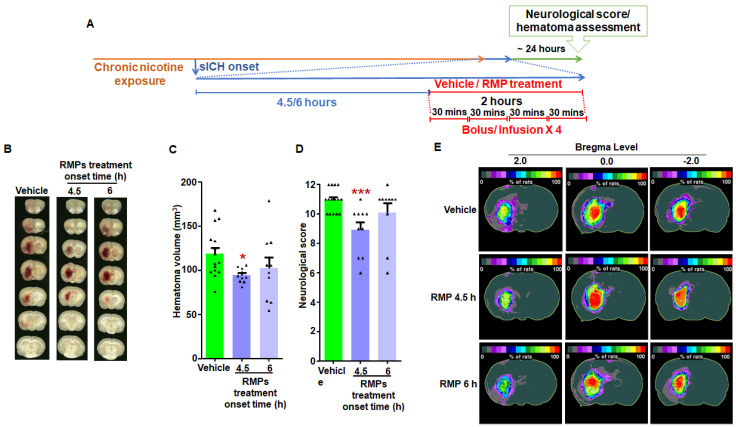
The effect of two different RMP treatment time points on hematoma expansion post-sICH in nicotine-exposed male rats: (**A**) representation of experimental design; (**B**) example images of brain scans showing mean hematoma volume; (**C**) mean hematoma volume data; (**D**) mean neurological score data; and (**E**) hematoma frequency maps at 3 coronal levels (bregma +2.0 mm, 0.0, and −2.0 mm). The top row depicts the hematoma frequency maps of vehicle-treated rats, the middle row demonstrates hematoma frequency maps of 4.5 h post-sICH RM-treated rats, and the bottom row shows the hematoma frequency maps of 6 h post-sICH RMP-treated rats. *N* = 15 for vehicle group, and *n* = 10 for RMP-treated groups. * *p* < 0.05 and *** *p* < 0.005 vs. vehicle group.

Given the extent of variation in time to reach a clinical facility for sICH patients [22,23], we ascertained the maximum therapeutic window of RMPs in limiting hematoma growth post-sICH in nicotine-exposed rats of both sexes. To define the therapeutic window, male and female rats were treated with either pooled vehicle (*n* = 10 and 5 for 4.5 and 6 h vehicle-treated groups, respectively) or RMPs starting at 4.5 (*n* = 10) or 6 h (*n* = 10) post-sICH induction. For male rats, the hematoma volume (mm^3^) for the 4.5 h (94 ± 3, *p* < 0.05) RMP treatment group was significantly lower than the respective pooled vehicle-treated group (119 ± 7) (Figure 3B,C). Similarly, the neurological score data in the 4.5 h (8.9 ± 0.5, *p* < 0.001) RMP treatment group was significantly lower than the respective pooled vehicle group (10.9 ± 0.2) (Figure 3D). However, the hematoma volume (mm^3^) (103 ± 12) and neurological score (10.1 ± 0.6) in the 6 h RMP treatment group were not significantly different from the respective pooled vehicle group (Figure 3B,C). Hematoma frequency maps also confirmed the hematoma volume results (Figure 3E).

**Figure 4:**
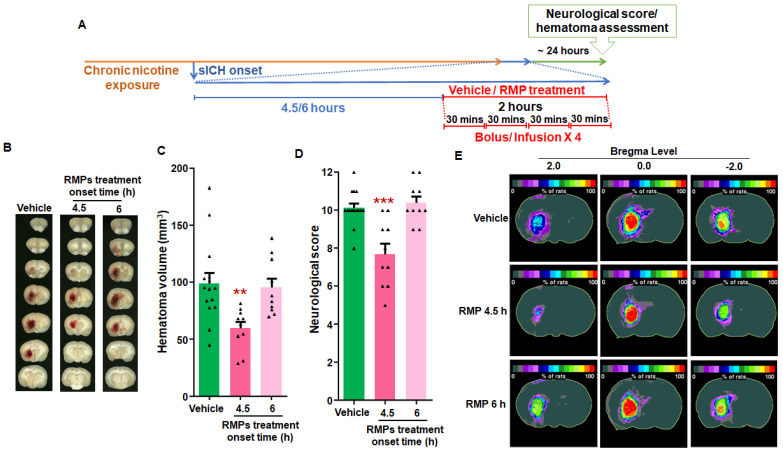
The effect of two different RMP treatment time points on hematoma expansion post-sICH in nicotine-exposed female rats: (**A**) representation of experimental design; (**B**) example images of brain scans showing mean hematoma volume; (**C**) mean hematoma volume data; (**D**) mean neurological score data; and (**E**) hematoma frequency maps at 3 coronal levels (bregma +2.0 mm, 0.0, to −2.00 mm). The top row depicts the hematoma frequency maps of vehicle-treated rats, the middle row demonstrates hematoma frequency maps of 4.5 h post-sICH RMP-treated rats, and the bottom row shows the hematoma frequency maps of 6 h post-sICH RMP-treated rats. *N* = 15 for vehicle group, and *n* = 10 for RMP-treated groups. ** *p* < 0.01 and *** *p* < 0.005 vs. vehicle group.

For female rats, the hematoma volume (mm^3^) for the 4.5 h (60 ± 6, *p* < 0.01) RMP treatment group was significantly lower than the respective pooled vehicle-treated group (99 ± 9) (Figure 4B,C). Similarly, the neurological score data in the 4.5 h (7.7 ± 0. 6, *p* < 0.001) RMP treatment group was significantly lower than the respective pooled vehicle group (10.1 ± 0.2) (Figure 4D). However, the hematoma volume (mm^3^) (96 ± 8) and neurological score (10.4 ± 0.3) in the 6 h RMP treatment group were not significantly different from those observed in the respective pooled vehicle group (Figure 4B,C). Hematoma frequency maps also support the hematoma volume results (Figure 4E). Our results show that RMPs limit hematoma expansion when administered as late as 4.5 h post-sICH in nicotine-exposed rats of both sexes.

### 2.5. The Effect of Treatment on Hematoma Growth in Male and Female Rats

The hematoma data of male and female groups for both experiments were compared. In terms of RMP efficacy data, we observed that the effect of RMP treatment (*p* < 0.005) as well as sex (*p* < 0.01) had a significant effect on hematoma volume. The hematoma volume in the vehicle-treated female group was 22% lower than that in the vehicle-treated male group. Moreover, since the hematoma volume in the female vehicle-treated group was lower, as expected, the hematoma volume in the RMP-treated female group was also lower (25%) than that observed in the RMP-treated male group. In terms of the therapeutic time window data, we observed that although the effect of RMP treatment was significantly different (*p* < 0.05), there was no statistically significant difference between the sexes (*p* = 0.06). Hematoma frequency maps supported the findings observed in terms of hematoma volume data (Figure 3). Hematoma appeared larger in both vehicle and RMP-treated male rats when compared to those from respective female groups. However, we did not observe any statistically significant difference between respective males and female groups.

## 3. Discussion

Tobacco use is one of the leading causes of death worldwide and is responsible for approximately 8 million deaths every year [24]. Moreover, in 2018, approximately 15% of adults have used e-cigarettes [25], and the potential ingredients that contribute to an adverse cardiovascular state with e-cigarette smoking include nicotine, among others [26,27,28]. sICH is a serious form of stroke without any effective therapy and is associated with a mortality rate of ≈50% within the first year of initial hospitalization [4]. The risk of sICH and worsening of outcomes following sICH is even higher in the smoking population in comparison to non-smokers [5,29]. The mortality rate in subjects with a larger hematoma expansion is higher than in subjects showing a smaller hematoma expansion [30,31], and thus limiting bleeding in the brain following sICH is promising as an effective therapeutic strategy. RMPs are new, potent hemostatic agents [15,32] that have shown efficacy in limiting hematoma growth and neurological impairment following sICH in naïve rats [18]. However, to ascertain the translational potential of a new drug for stroke treatment, guidelines suggest that besides testing the efficacy in normal animals, it is also essential to test the efficacy in animal models of other comorbid conditions and risk factors [33]. Since tobacco use is a risk factor for sICH [5,19,20], testing the efficacy of RMPs in an animal model of tobacco use would further support its applicability in a clinical setting. Therefore, we tested the potential effect of RMPs on hematoma growth following sICH in nicotine-exposed rats. Our results demonstrate that RMP treatment limits hematoma volume and neurological impairment post-sICH in rats of either sex exposed to chronic nicotine administration.

Nicotine is shown to affect blood coagulation. Generally, cigarette smoking is reported to activate platelets and cause enhanced procoagulant activity [34,35,36,37]. Moreover, cigarette smoke extracts compromise the cellular integrity of endothelial cells and increase oxidative stress [38]. While high nicotine concentrations retard the clotting mechanism of thrombin, lower concentrations accelerate clot formation [39]. These observations are based on the acute effects of nicotine and test the effect in a concentration of nicotine higher than the levels attainable in the present study. Our previous study demonstrated that chronic nicotine exposure, as employed in the present study, causes enhanced bleeding in the brain following sICH [8]. These differences in blood coagulation parameters in the presence and absence of nicotine/tobacco exposure further underscore the importance of testing the efficacy of RMPs in limiting hematoma growth in an animal model of tobacco use.

On the basis of the pharmacokinetic data of RMPs [32], we have previously compared the efficacy of different treatment regimens of RMPs in sICH-induced naïve rats. We employed the optimal paradigm observed in that study for our present study [18]. We also observed that RMP treatment did not produce any acute toxic effects on rats [40]. Further, in the present study, we observed that RMP treatment did not have a significant impact on mortality and physiological functions determined in terms of blood gases ≈24 h after treatment. In human studies, hematoma expansion decreases 3–6 h after sICH onset [41]. Moreover, the time taken between sICH onset and the arrival of the patient at a healthcare facility for potential therapy varies considerably [22,42]. Nevertheless, owing to the lack of effective treatment options, the clinical outcome even following improved hospital arrival time post-sICH has failed to improve outcomes in sICH subjects [43]. Therefore, it is critical to identify new therapies that are effective after the subjects reach a clinical facility [33]. We have previously observed that RMPs limit hematoma expansion in naïve rats when administered up to 4.5 h after sICH onset [18]. In line with our previous observations, we found that RMPs also limit hematoma expansion in nicotine-exposed rats when administered as late as 4.5 h after sICH. Therefore, RMPs appear to have a clinically relevant therapeutic effect window that could qualify for treatment of a large subset of smokers who suffer from sICH.

In the present study, we observed that RMPs limit hematoma growth and neurological impairment when assessed 24 h post-sICH when treated with RMPs starting up to 4.5 h post-sICH. However, guidelines also recommend the evaluation of multiple endpoints such as long-term histological and behavioral outcomes post-sICH to verify the sustained efficacy of new therapeutic agents [33]. Further, other recommendations include replication of the efficacy studies and testing the efficacy in aged animals [34]. On the lines of the above guidelines, more studies are required in the future to affirm the efficacy of RMPs to treat sICH in nicotine-treated rats.

Some of the literature has compared sex differences in sICH outcomes. One study demonstrated that men are at greater risk of death after one month of sICH when compared to women [44]. Moreover, there is an increased chance of hematoma expansion in males versus females [45]. The relationship between in-hospital mortality in men with sICH was stronger than in women [46], while another study showed that females have higher chances of worse outcomes and mortality post-sICH than males [47]. However, female mice demonstrate a lower extent of edema and an increased improvement in behavioral deficits post-sICH when compared to male mice [48]. In addition, we have previously observed that the hematoma volume in nicotine- and vehicle-exposed males was higher than that in the female rats. Similarly, in the present study, we observed that the hematoma in vehicle- and RMP-treated male rats was higher than that observed in female rats. We also observed a similar trend in the 4.5 h RMP-treated groups between both sexes. There was a non-significant decreasing trend in hematoma volume in male rats treated with RMPs 6 h post-sICH. A similar non-significant lower decreasing trend was observed in female rats treated with RMPs 6 h post-sICH. The experiments on male and female rats were not conducted simultaneously. Therefore, we cannot make a direct comparison of male and female groups. Studying male and female groups in parallel may help confirm potential sex differences, if any.

In comparison to males, the pharmacokinetics of drugs in females are altered by some physiological differences such as lower body weight, hepatic biotransformation, and slower glomerular filtration rate [49,50]. Moreover, owing to certain pharmacodynamic causes, some drugs exert different extents of efficacy in men versus women [51]. Therefore, it is important to assess the efficacy of novel drugs in both males and females [52]. In the present study, we observed that RMPs limit hematoma growth in both nicotine-exposed male and female rats.

## 4. Methods

### 4.1. Animals

The experiments in the present study were carried out in accordance with the guidelines laid down by the National Institutes of Health in the Guide for the Care and Use of Laboratory Animals. The experimental protocol was approved by the University of Miami Institutional Animal Care and Use Committee. Young Sprague Dawley rats of both sexes were obtained from Charles River Laboratories (Wilmington, MA, USA). Group randomization, predetermined sample size estimates based on power analysis of preliminary data, predefined exclusion criteria, and blinded analyses were employed to ensure rigor in the present study.

### 4.2. Nicotine Administration

Chronic nicotine exposure was carried out using subcutaneously implanted osmotic pumps (DURECT Corporation, Cupertino, CA, USA), infusing nicotine bis-L-(+)-tartrate dehydrate (4.5 mg/kg b.w./day) over an average period of 14.8 ± 0.2 days (considered as a period of chronic exposure). The dose and regimen of nicotine exposure were selected to maintain the levels of nicotine and cotinine as observed in chronic smokers [53,54,55].

### 4.3. Production of RMPs

O+ leukoreduced RBCs (OneBlood Center, Ft. Lauderdale, FL) were washed with saline [15] and passed through a high-pressure extrusion system (Constant System Cell Disruptor, Northants, UK) two times at a pressure of 35,000 psi to generate RMPs [33]. The RMPs were then washed with normal saline and stored with a preservative (0.005% thimerosal) at −80 °C. Before use, RMPs were vortexed and sonicated three times for 1 s each. The RMP concentration and procoagulant activity were determined in each batch using flow cytometer-based analysis and thromboelastography, respectively [15,32].

### 4.4. Monitoring of the Stage of the Estrous Cycle

Female patients requiring treatment for sICH may present themselves in the clinical setting irrespective of the stage of their menstrual cycle. Therefore, we used a mixed population of rats in different stages of their estrous cycle. Study groups were balanced in terms of the proportion of female rats in specific stages of the estrous cycle to account for the potentially variable effect of treatment(s) on rats at different stages of the estrous cycle. Stages of the estrous cycle in the rats were monitored as described previously [56]. Vaginal smears were prepared and examined microscopically to identify the stage of the estrous cycle prior to sICH induction. Given that the period of metestrus is very short, and that the hormonal changes associated with metestrus are like diestrus and those of estrus are like proestrus, we grouped the data of animals in metestrus with the diestrus stage and the data of animals in proestrus with the estrus stage [56].

### 4.5. sICH Induction

Rats were weighed and their blood glucose levels were monitored prior to surgery. The animals were anesthetized with isoflurane in a mixture of oxygen and nitrous oxide (30:70 ratio). The femoral artery was cannulated to allow continuous blood pressure monitoring and blood sampling for the determination of blood gases. The femoral vein was cannulated to administer rocuronium and the specified treatment (vehicle or RMPs). The rats were then intubated, paralyzed, and put on a mechanical ventilator. Physiological parameters (head and body temperature, blood pH, partial pressure of carbon dioxide (pCO_2_), partial pressure of oxygen (pO_2_) in the blood, and mean arterial blood pressure (MABP) were monitored and maintained within a normal range before and after collagenase injection, as well as during vehicle/RMP treatment. The rats were then placed on a stereotaxic frame, and a ≈2 cm long sagittal incision was made over the skull. The tissue over the skull was cleared to identify the bregma, and a small burr hole was made (3.2 mm medio-lateral and 0.2 mm antero-posterior to the bregma) using a drill on the right aspect of the skull. Using a drill and injection robot (Neurostar, Tubingen, Germany), a 30-G needle connected to a Hamilton syringe (10 μL capacity, Hamilton Company, Reno, NV, USA) was inserted 3.2 mm to the right, 6.5 mm deep, and 0.2 mm anterior to the bregma at a rate of 0.2 mm/s, and 0.12 U of collagenase was injected into the striatum [21,57]. A total of 2.0 μL collagenase solution was infused over 5 min. The needle was allowed to stay at the site of injection for an additional 5 min to facilitate diffusion of the solution into the brain tissue and avoid sudden leakage via the hole in the brain. The syringe was then removed, and the hole was sealed with bone wax. Sutures were used to close the skin, and proper post-operative care was given to the animal.

### 4.6. Determination of Neurological Score

Neurological impairment was determined ≈24 h after induction of sICH using the neurological score explained previously [58]. This measure involved the assessment of postural reflex, tactile placing, visual placing, and proprioceptive placing tests. The score varied from 0 and 12, indicating increasing levels of neurological impairment with an increase in score.

### 4.7. Animal Perfusion and Brain Isolation

Rats were anesthetized with isoflurane in a mixture of oxygen and nitrous oxide (30:70 ratio) ≈24 h after collagenase injection. A median sternotomy was carried out, and a cut was made in the apex of the heart’s left ventricle. Polyethylene tubing was gently inserted into the aorta via the heart and fastened in place with a suture. A cut in the right auricle was made to allow the outflow of the perfusate. The rats were then perfused with cold normal saline at a pressure of ≈120 mm Hg for a period of 2–3 min to ensure the removal of all blood from the rats’ bodies. The rats were then decapitated, and their brains were isolated.

### 4.8. Assessment of Hematoma Volume

The brain was then placed on a rat brain matrix and hardened by placing it in the −80 °C freezer for a period of ≈5 min. The brain was then sectioned using single-edge industrial razor blades into coronal slices of 2 mm thickness. The rostral side of the sections was considered side A, and the caudal side of the sections was considered side B. The bregma levels represented by side A brain sections were from +6 mm to −6 mm with a consecutive gap of 2 mm each. The slices were scanned at a resolution of 24-bit true color per pixel and 2400 dots per inch. The images were checked for the presence of artifacts, and acceptable scans were analyzed in a blinded manner using ImageJ software. The hematoma area calculated from the scans of both sides was averaged, and the hematoma volume for each section was calculated. The total hematoma volume was obtained by adding values of each section [57].

Scans of each section were further processed. Image mapping for each group was performed using an established computer program for lesion frequency distribution analysis [59]. In brief, each section of the brain was mapped into its corresponding digital rat brain atlas. Two distinct intensity values were assigned to regions classified as hematoma and normal brain tissue, separately. Rats in the same group were then averaged for each corresponding bregma level. The normalized averaged image was the frequency distribution map of hematoma. Each treatment group processed from scans of side A yields the occurrence of the hematoma on a pixel basis. The resulting processed images were mirror images of the original scans, and thus the hematoma in frequency maps is shown as being on the opposite side of the brain.

### 4.9. Experimental Protocol

Given the different periods of anesthesia in different treatment groups, control animals (vehicle-treated group) similarly treated with anesthesia were employed. Randomization was carried out after sICH induction and prior to the treatment.

Experiment 1: To determine the efficacy of RMP treatment in limiting hematoma volume following sICH in nicotine-exposed rats, vehicle- (saline) and RMP-treated groups were employed. The treatment started 2 h after collagenase injection with 1/3 dose administered as a bolus injection and the remaining amount infused over 29 min to a total of 3.34 × 10^10^ particles/kg b.w. Each rat received four such doses of RMPs (total duration of 2 h) [18]. A similar set of groups was employed for male and female rats (Figure 1A and Figure 2A).

Experiment 2: To determine the maximum therapeutic window of RMPs in preventing hematoma growth following sICH in nicotine-exposed rats, one vehicle-treated and two RMP-treated groups were employed. Vehicle treatment started at either 4.5 or 6 h post-collagenase injection in the pooled vehicle-treated control group. RMP treatment started at either 4.5 or 6 h post-collagenase injection [18]. A similar set of groups was employed for male and female rats (Figure 3A and Figure 4A). In the RMP treatment groups, each rat received a total of four doses of RMPs over a 2 h period, as described in experiment 1. Each dose consisted of 3.34 × 10^10^ particles/kg b.w. for the 4.5 h treatment groups in both male and female rats, as well as 6 h treatment groups in female animals. However, the 6 h treatment group of male rats received a dose of 3.96 × 10^10^ particles/kg b.w. The 6 h RMP treatment group received a slightly higher dose than other RMP treatment groups, owing to the batch-to-batch variation of RMPs used in this group.

To mimic the clinical situation of patients who are unable to smoke after hospitalization due to sICH, the osmotic pump administering nicotine was removed prior to the onset of sICH.

### 4.10. Statistical Analysis

GraphPad Prism software version 5 was employed to perform statistical analyses. Grubbs’ test was used to identify statistically significant outlier data points, which were excluded from further analysis (1 male rat in the 4.5 h post-sICH RMP treatment group was excluded because its hematoma volume data point was an outlier). Data of different groups were compared using Student’s *t*-test. The hematoma volume of male and female groups for both experiments was compared using two-way ANOVA. A chi-squared test was performed to compare the distribution of percentage animals in different stages of the estrous cycle. A *p*-value of <0.05 was considered statistically significant. The results are expressed as mean ± SEM.

**Figure 5:**
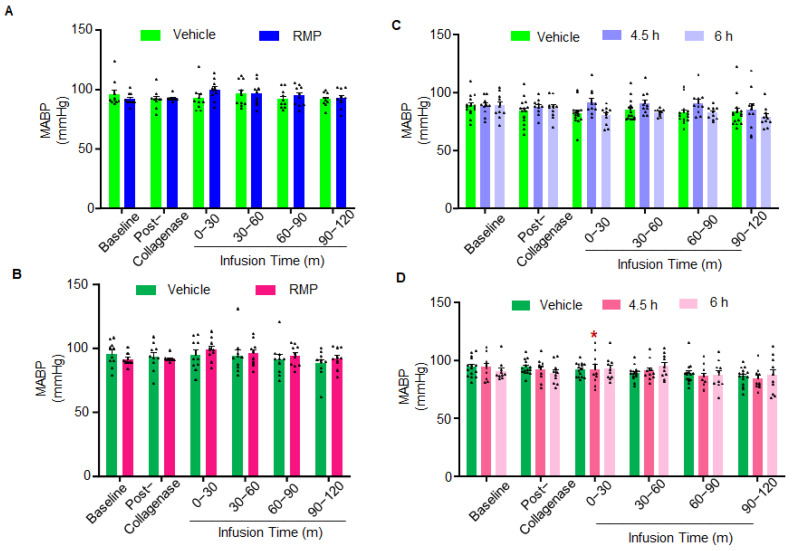
MABP data of groups of (**A**) male rats receiving vehicle or RMP; (**B**) female rats receiving vehicle or RMP; (**C**) male rats receiving vehicle or RMP 4.5 and 6 h post-sICH; and (**D**) female rats receiving vehicle or RMP 4.5 and 6 h post-sICH. * *p* < 0.05 vs. vehicle-treated rats.

The following exclusion criteria were defined prior to the study: (1) rats with a body weight above 375 g (none); (2) rats with a mean MABP continuously for 10 min above 135 or below 80 mmHg before and after collagenase injection (2 vehicle-treated female rats treated 2 h post-sICH; Figure 5A−D); (3) leaky or blocked syringe needle used for the collagenase injection (none); (4) rats that did not receive the collagenase injection (before, during, and after injection) at the intended site in the brain (1 vehicle-treated male and female rat treated 2 h post-sICH; 2 RMP-treated female rats treated 2 h post-sICH; 2 vehicle-treated female and 1 RMP-treated female rat treated 6 h post-sICH; 1 RMP-treated male and female rat treated 4.5 h post-sICH); (5) rats with blood glucose levels above 160 mg/dL (none); (6) rats with surgical complications such as severe blood loss or blockade in the vein or artery catheter (1 RMP-treated female rat treated 4.5 h post-sICH); and (7) rats with blood gases outside the normal range (none). Excluded animals were replaced with additional animals to meet the sample size requirements per power analysis. In addition, the brain slices from bregma level 6.0 for two vehicle-treated male and female rats from Experiment 1 were excluded from the hematoma frequency distribution analysis because the bregma levels of these brain slices did not match with the other animals. These animals were, however, included in hematoma analysis using ImageJ.

## 5. Conclusions

Overall, we conclude that RMP treatment can limit hematoma growth and attenuate neurological impairment post-sICH in both sexes in an animal model of tobacco use, a prominent risk factor for sICH. We further demonstrate that RMPs limit hematoma volume when administered as late as 4.5 h after the onset of sICH in male and female rats previously exposed to nicotine. Therefore, we infer that RMPs have the potential to limit hematoma growth in sICH patients who are using tobacco products.

## Figures and Tables

**Table 1:** Physiological parameters before, during, and after collagenase injection and during vehicle/RMP treatment in male rats employed in the efficacy experiment.

Groups	Sampling Time	Body Weight (g)	Body Temperature (°C)	Head Temperature (°C)	pH	pCO_2_ (mmHg)	pO_2_ (mmHg)	MABP (mmHg)	Blood Glucose (mg/dL)
Vehicle-treated (*n* = 10)	Before	297 ± 3	36.8 ± 0.1	36.6 ± 0.0	7.30 ± 0.01	37 ± 1	113 ± 7	98 ± 3	135 ± 8
	During		36.9 ± 0.1	36.6 ± 0.0	7.31 ± 0.01	36 ± 1	110 ± 6	94 ± 2	
	0.5 h after		36.9 ± 0.1	36.8 ± 0.1	7.31 ± 0.01	36 ± 0	108 ± 5	97 ± 2	
	1 h after		37.2 ± 0.1	36.9 ± 0.1	7.29 ± 0.01	38 ± 1	122 ± 6	101 ± 3	
	3 h after		37.2 ± 0.1	36.8 ± 0.1	7.30 ± 0.01	36 ± 1	110 ± 5	98 ± 2	
	3.5 h after		37.1 ± 0.1	36.8 ± 0.1	7.29 ± 0.01	37 ± 1	108 ± 7	96 ± 2	
	24 h after		37.3 ± 0.1	36.7 ± 0.0	7.30 ± 0.01	40 ± 0	124 ± 6	101 ± 5	
RMP- treated (*n* = 10)	Before	299 ± 7	36.7 ± 0.1	36.9 ± 0.1 **	7.31 ± 0.01	38 ± 1	95 ± 3 *	97 ± 2	134 ± 8
	During		36.8 ± 0.1	36.7 ± 0.1	7.31 ± 0.01	38 ± 1	95 ± 4	98 ± 2	
	0.5 h after		36.9 ± 0.1	36.8 ± 0.1	7.32 ± 0.01	36 ± 0	105 ± 5	98 ± 2	
	2 h after		36.9 ± 0.1	36.9 ± 0.0	7.29 ± 0.01	37 ± 1	121 ± 6	98 ± 3	
	3 h after		36.8 ± 0.1 **	36.8 ± 0.1	7.30 ± 0.01	37 ± 0	124 ± 6	98 ± 2	
	3.5 h after		37.0 ± 0.1	36.8 ± 0.1	7.30 ± 0.00	36 ± 1	123 ± 9	97 ± 2	
	24 h after		37.2 ± 0.1	36.7 ± 0.1	7.31 ± 0.01	39 ± 1	124 ± 3	100 ± 3	

* *p* < 0.05 vs. respective vehicle control. ** *p* < 0.01 vs. respective vehicle control.

**Table 2:** Physiological parameters before, during, and after collagenase injection and during vehicle/RMP treatment in female rats employed in the efficacy experiment.

Groups	Sampling Time	Body Weight (g)	Body Temperature (°C)	Head Temperature (°C)	pH	pCO_2_ (mmHg)	pO_2_ (mmHg)	MABP (mmHg)	Blood Glucose (mg/dL)
Vehicle-treated (*n* = 10)	Before	272 ± 3	36.9 ± 0.1	36.7 ± 0.1	7.28 ± 0.01	35 ± 1	114 ± 5	96 ± 2	127 ± 4
	During		36.9 ± 0.1	36.7 ± 0.0	7.28 ± 0.01	35 ± 1	109 ± 5	97 ± 2	
	0.5 h after		37.0 ± 0.1	36.9 ± 0.1	7.28 ± 0.01	36 ± 1	109 ± 5	101 ± 1	
	2 h after		37.0 ± 0.1	36.8 ± 0.1	7.25 ± 0.01	37 ± 1	119 ± 7	102 ± 3	
	3 h after		37.0 ± 0.1	36.9 ± 0.1	7.25 ± 0.01	36 ± 1	120 ± 6	98 ± 3	
	3.5 h after		37.0 ± 0.1	36.9 ± 0.1	7.26 ± 0.01	36 ± 0	118 ± 6	93 ± 2	
	24 h after		37.1 ± 0.1	36.7 ± 0.0	7.27 ± 0.01	37 ± 1	123 ± 5	92 ± 2	
RMP- treated (*n* = 10)	Before	285 ± 5	36.9 ± 0.1	36.6 ± 0.1	7.27 ± 0.01	37 ± 1	103 ± 10	96 ± 3	138 ± 6
	During		36.9 ± 0.1	36.9 ± 0.1	7.26 ± 0.01 *	38 ± 1 *	104 ± 8	98 ± 2	
	0.5 h after		37.0 ± 0.1	36.8 ± 0.1	7.28 ± 0.00	37 ± 0	110 ± 9	100 ± 2	
	2 h after		36.9 ± 0.1	36.8 ± 0.1	7.27 ± 0.01	37 ± 1	112 ± 6	98 ± 2	
	3 h after		37.0 ± 0.1	37.0 ± 0.1	7.27 ± 0.01	36 ± 1	106 ± 5	101 ± 3	
	3.5 h after		37.0 ± 0.1	37.0 ± 0.1	7.26 ± 0.01	37 ± 1	108 ± 8	98 ± 2	
	24 h after		37.1 ± 0.1	36.8 ± 0.1	7.27 ± 0.01	37 ± 1	116 ± 8	88 ± 2	

* *p* < 0.05 vs. respective vehicle control.

**Table 3:** Physiological parameters before, during, and after collagenase injection and during vehicle/RMP treatment in male rats employed in the therapeutic window experiment.

Groups	Sampling Time	Body Weight (g)	Body Temperature (°C)	Head Temperature (°C)	pH	pCO_2_ (mmHg)	pO_2_ (mmHg)	MABP (mmHg)	Blood Glucose (mg/dL)
Vehicle-treated (*n* = 15)	Before	315 ± 4	36.8 ± 0.1	36.7 ± 0.0	7.46 ± 0.01	37 ± 1	105 ± 4	96 ± 1	122 ± 4
	During		36.8 ± 0.1	36.6 ± 0.1	7.47 ± 0.01	36 ± 1	103 ± 4	93 ± 2	
	0.5 h after		37.0 ± 0.1	36.8 ± 0.1	7.48 ± 0.00	34 ± 0	103 ± 3	91 ± 2	
	4.5 h after (*n* = 10)		37.0 ± 0.1	36.7 ± 0.1	7.43 ± 0.01	39 ± 1	120 ± 7	92 ± 2	
	5.5 h after (*n* = 10)		36.9 ± 0.1	36.7 ± 0.1	7.42 ± 0.01	38 ± 1	114 ± 8	91 ± 1	
	6 h after (*n* = 5)		37.1 ± 0.2	36.9 ± 0.2	7.44 ± 0.01	39 ± 1	104 ± 3	98 ± 3	
	6.5 h after (*n* = 10)		37.0 ± 0.1	36.8 ± 0.1	7.43 ± 0.01	37 ± 1	110 ± 5	89 ± 1	
	7 h after (*n* = 5)		37.3 ± 0.1	36.9 ± 0.2	7.45 ± 0.00	37 ± 1	94 ± 2	98 ± 2	
	8 h after (*n* = 5)		37.3 ± 0.1	37.0 ± 0.2	7.45 ± 0.00	37 ± 1	98 ± 2	100 ± 4	
	24 h after		37.3 ± 0.1	36.7 ± 0.0	7.46 ± 0.01	36 ± 1	112 ± 5	88 ± 1	
4.5 h window (*n* = 10)	Before	315 ± 5	36.8 ± 0.1	36.6 ± 0.0	7.46 ± 0.01	37 ± 1	100 ± 7	98 ± 2	120 ± 5
	During		36.9 ± 0.1	36.7 ± 0.1	7.47 ± 0.01	36 ± 1	99 ± 7	95 ± 2	
	0.5 h after		37.0 ± 0.1	36.8 ± 0.1	7.47 ± 0.01	36 ± 1 *	103 ± 6	93 ± 2	
	4.5 h after		37.0 ± 0.1	37.0 ± 0.1	7.45 ± 0.01	37 ± 1	112 ± 6	96 ± 3	
	5.5 h after		37.0 ± 0.1	37.1 ± 0.1 **	7.43 ± 0.01	37 ± 1	103 ± 6	96 ± 2	
	6 h after		37.0 ± 0.1	37.0 ± 0.1 *	7.44 ± 0.01	37 ± 1	103 ± 6	91 ± 2	
	24 h after		37.3 ± 0.2	36.7 ± 0.1	7.46 ± 0.01	37 ± 1	108 ± 7	91 ± 1	
6 h window (*n* = 10)	Before	310 ± 3	36.9 ± 0.1	36.7 ± 0.1	7.47 ± 0.01	36 ± 1	103 ± 6	94 ± 2	107 ± 7
	During		36.8 ± 0.1	36.6 ± 0.1	7.47 ± 0.01	36 ± 1	104 ± 6	94 ± 3	
	0.5 h after		37.0 ± 0.1	36.7 ± 0.1	7.47 ± 0.01	35 ± 0	103 ± 5	94 ± 2	
	6 h after		37.0 ± 0.1	36.8 ± 0.1	7.42 ± 0.01	38 ± 1	129 ± 8	94 ± 3	
	7 h after		37.2 ± 0.1	37.0 ± 0.1	7.42 ± 0.01 *	38 ± 0	113 ± 5	94 ± 2	
	8 h after		37.1 ± 0.1	36.8 ± 0.1	7.42 ± 0.01 **	38 ± 0	112 ± 6	89 ± 2	
	24 h after		37.5 ± 0.1	36.8 ± 0.0	7.45 ± 0.01	36 ± 1	123 ± 7	89 ± 2	

* *p* < 0.05 vs. respective vehicle control. ** *p* < 0.01 vs. respective vehicle control. Different sets of animals that received vehicle treatment after different time intervals post-sICH were pooled together, and physiological parameters were measured at different time points depending on the experiments. Therefore, n-values for the vehicle group are different for different time points.

**Table 4:** Physiological parameters before, during, and after collagenase injection and during vehicle/RMP treatment in female rats employed in the therapeutic window experiment.

Groups	Sampling Time	Body Weight (g)	Body Temperature (°C)	Head Temperature (°C)	pH	pCO_2_ (mmHg)	pO_2_ (mmHg)	MABP (mmHg)	Blood Glucose (mg/dL)
Vehicle-treated (*n* = 15)	Before	248 ± 3	36.9 ± 0.1	36.7 ± 0.1	7.37 ± 0.03	38 ± 1	103 ± 4	98 ± 1	113 ± 3
	During		36.9 ± 0.1	36.7 ± 0.1	7.37 ± 0.02	37 ± 0	104 ± 4	97 ± 2	
	0.5 h after		37.0 ± 0.1	36.8 ± 0.1	7.38 ± 0.03	35 ± 1	104 ± 3	99 ± 1	
	4.5 h after (*n* = 10)		37.1 ± 0.1	36.7 ± 0.1	7.32 ± 0.02	38 ± 1	117 ± 8	99 ± 1	
	5.5 h after (*n* = 10)		37.2 ± 0.1	36.8 ± 0.1	7.33 ± 0.03	36 ± 1	110 ± 6	93 ± 2	
	6 h after (*n* = 5)		36.9 ± 0.2	36.7 ± 0.1	7.45 ± 0.01	36 ± 1	96 ± 6	97 ± 4	
	6.5 h after (*n* = 10)		37.2 ± 0.1	36.8 ± 0.1	7.32 ± 0.00	36 ± 1	110 ± 3	91 ± 1	
	7 h after (*n* = 5)		36.9 ± 0.1	36.7 ± 0.1	7.45 ± 0.01	37 ± 1	96 ± 5	99 ± 3	
	8 h after (*n* = 5)		37.1 ± 0.2	37.0 ± 0.1	7.44 ± 0.01	36 ± 1	91 ± 4	97 ± 1	
	24 h after		37.4 ± 0.1	36.7 ± 0.1	7.38 ± 0.02	37 ± 1	115 ± 5	94 ± 2	
4.5 h window (*n* = 10)	Before	258 ± 6	36.9 ± 0.1	36.8 ± 0.1	7.35 ± 0.01	37 ± 0	107 ± 9	98 ± 2	119 ± 5
	During		37.0 ± 0.1	36.7 ± 0.1	7.35 ± 0.02	36 ± 1	111 ± 9	98 ± 2	
	0.5 h after		37.2 ± 0.1	36.8 ± 0.1	7.36 ± 0.03	35 ± 1	108 ± 8	96 ± 2	
	4.5 h after		37.0 ± 0.1	37.0 ± 0.1 *	7.35 ± 0.03	36 ± 1	126 ± 8	97 ± 3	
	5.5 h after		37.1 ± 0.1	37.1 ± 0.1	7.34 ± 0.03	35 ± 1	116 ± 6	93 ± 2	
	6.5 h after		37.0 ± 0.1	36.8 ± 0.1	7.35 ± 0.03	35 ± 1	112 ± 5	95 ± 2	
	24 h after		37.3 ± 0.2	36.7 ± 0.0	7.37 ± 0.04	36 ± 1	110 ± 6	92 ± 1	
6 h window (*n* = 10)	Before	253 ± 5	36.8 ± 0.1	36.7 ± 0.1	7.45 ±0.01 *	37 ± 1	103 ± 5	96 ± 1	106 ± 3
	During		36.8 ± 0.1	36.7 ± 0.0	7.45 ± 0.01	38 ± 1	103 ± 4	95 ± 2	
	0.5 h after		37.0 ± 0.1	36.7 ± 0.1	7.48 ±0.01 **	35 ± 1	99 ± 3	94 ± 2	
	6 h after		37.0 ± 0.1	36.8 ± 0.1	7.46 ± 0.01	37 ± 1	114 ± 7	96 ± 2	
	7 h after		37.0 ± 0.1	36.9 ± 0.1	7.46 ± 0.01	36 ± 1	111 ± 5	97 ± 2	
	8 h after		37.2 ± 0.1	37.0 ± 0.1	7.44 ± 0.01	37 ± 1	106 ± 4	93 ± 2	
	24 h after		37.8 ± 0.1 **	36.8 ± 0.1	7.47 ± 0.01 *	37 ± 1	104 ± 4	95 ± 2	

* *p* < 0.05 vs. respective vehicle control. ** *p* < 0.01 vs. respective vehicle control. Different sets of animals that received vehicle treatment after different time intervals post-sICH were pooled together, and physiological parameters were measured at different time points depending on the experiments. Therefore, n-values for the vehicle group are different for different time points.

## Data Availability

All data generated or analyzed during this study are included in this article.
